# A Machine Learning Approach for the Automatic Estimation of Fixation-Time Data Signals’ Quality

**DOI:** 10.3390/s20236775

**Published:** 2020-11-27

**Authors:** Giulio Gabrieli, Jan Paolo Macapinlac Balagtas, Gianluca Esposito, Peipei Setoh

**Affiliations:** 1 Psychology Program, School of Social Sciences, Nanyang Technological University, Singapore 639818, Singapore; GIULIO001@e.ntu.edu.sg (G.G.); JANP0001@e.ntu.edu.sg (J.P.M.B.); gianluca.esposito@ntu.edu.sg (G.E.); 2 Lee Kong Chian School of Medicine, Nanyang Technological University, Singapore 636921, Singapore; 3 Department of Psychology and Cognitive Science, University of Trento, 38068 Rovereto, Italy

**Keywords:** eye-tracking, machine learning, signal quality

## Abstract

Fixation time measures have been widely adopted in studies with infants and young children because they can successfully tap on their meaningful nonverbal behaviors. While recording preverbal children’s behavior is relatively simple, analysis of collected signals requires extensive manual preprocessing. In this paper, we investigate the possibility of using different Machine Learning (ML)—a Linear SVC, a Non-Linear SVC, and K-Neighbors—classifiers to automatically discriminate between *Usable* and *Unusable* eye fixation recordings. Results of our models show an accuracy of up to the 80%, suggesting that ML tools can help human researchers during the preprocessing and labelling phase of collected data.

## 1. Introduction

Developmental studies, particularly those with preverbal young children, pose a unique challenge in that participants are unable to provide an oral account of their decision or preferences. Noninvasive techniques which do not require verbal abilities, such as pupil dilation and fixation time measures, have been successfully employed in studies with infants and young children to gain insight into their cognition [1,2,3,4,5,6]. One looking time method taps on young children’s spontaneous responses to events which violate their expectations given their understanding of the world [7,8,9]. In Setoh et al. [6], for example, eight-month-old infants’ looking time was used as a measure to investigate their abstract biological expectations about animals. The violation-of-expectation (VOE) paradigm has also been used to examine infants’ understanding of basic numeracy [10], false beliefs [11], and even whether objects can fly [12]. This paradigm has even been successfully utilised in infant studies on newborns as young as four days old [13]. The VOE framework is based on the premise that young children tend to fixate at events that violate their expectation for a longer amount of time than at events that do not. In both adult [14] and infant [15] populations, contemporary researchers have used eye-tracking technology to measure changes in the eyes, such as fixation timing and pupil dilation, in response to experimental tasks. For instance, pupil dilation and constriction in adult participants varied as a function of the presentation of pictures with different emotional valence [16]. Moreover, a recent review by Moch et al. [17] found that duration, as well as location of eye fixations can be reliable indices of underlying numerical processing in adults. Employing methods based on habituation and VOE, researchers have been able to investigate preverbal children’s moral understanding. Similar methods have been employed to show that preverbal toddlers disapprove of disgusting acts [18], show prosocial behavior [19,20], differentiate between good and evil [21,22], and display empathy and compassion [23].

Despite the benefits of the approach, fixation time paradigms present some limitations. In a recent ethnographic study, Peterson [24] discussed the problems related to the VOE paradigm. In VOE experiments, infants or young children’s attention is typically measured as the interval of time that occurs between the offset of presentation of a stimuli to the moment in which the infant or child loses interest towards the scene [5]. Children’s looking time at the scene is used as an indicator of the attention [25]. Although eye-tracking devices can be employed to automatically record looking time, a visual inspection of recorded videos is usually performed to manually verify the usability of a sample. This is because testing children presents its own set of challenges. While it is possible to ask adult participants to stay still after the eye-tracking machine has been calibrated, the same cannot be expected for infants and toddlers. For example, a child may look away frequently because he is distracted, but the sample will be reported by the eye-tracking device as acceptable if each fixation away from the screen is shorter than a preset look-away threshold, which is usually set at 2 s [5]. Another scenario could be that a sample may be reported as *unusable* if the child moved away from the eye-tracker calibration point despite being attentive. The manual inspection stage is generally performed by trained researchers and is often conducted by multiple individuals in order to ascertain observer reliability. The problem with manual inspection and selection of samples after data collection is not only in the additional time incurred but also that these judgements of what makes a sample *usable* or *unusable* may not be reproducible or consistent across different studies or different laboratories [24,26]. As pointed out by Peterson [24], working with young children requires researchers to practice constant and active decision making, in both the execution of the experiment and coding of the children’s behavioural recordings. Despite the wide adoption of the VOE paradigm in infant and child studies, standardised impartial methods to code for inclusion or exclusion of samples based on behavioural measures have not been developed.

Previous works have shown that Machine Learning (ML) models can be successfully employed to study neurophysiological signals. In Gabrieli et al. [27], for example, different machine learning models were tested to verify the possibility of classifying infants’ vocalisations while in other works the technique was shown to be suitable for the automatic identification of physiological signal quality [28,29]. Li et al. [29], for example, employed Support Vector Machines (SVM) models to distinguish between clean and noisy electrocardiogram (ECG) recording. For what concerns SVMs application to eye-tracking, Pauly et al. Pauly and Sankar [30] demonstrated the suitability of the models for the detection of drowsiness from adults’ eye-tracked blinks. Similarly, Kang et al. [31] employed SMV classifiers to distinguish between autistic versus typically developing three to six year old children using eye-tracking and EEG recordings. In an analogue way, different studies employed a Linear SVM for different classification tasks involving eye-tracking data. Dalrymple et al. [32], for example, successfully employed Linear SVM classifiers on eye-tracking data to identify the age of toddlers, while Wang et al. [33] used Linear SVM, in combination with gaze data to quantify atypical visual saliency in children diagnosed with autism spectrum disorder. Linear and Non-Linear SVM are not the only classifiers that have been successfully employed on eye-tracking data. K-nearest neighbor (kNN) classifiers have been widely employed to make classification out of eye-tracking and gazing data. Zhang et al. [34], Kacur et al. [35] used a kNN classifier to diagnose schizophrenia disorders using an eye-tracking system during Rorschach Inkblot Test [36].

It is therefore possible that ML models can be employed to objectively classify the usability of infants’ and young children’s fixation time recordings, and in doing so, reduce the subjectivity of behavioural codings as decided by researchers themselves.

Here we aim at testing three different classifiers: a Linear SVM, a Non-Linear SVM, and a kNN classifiers. Our method involves a binary classification task, where data of one trial have to be classified as usable or unusable. For each trial, three repetitions are conducted with each child, therefore resulting in a dataset with a small number of features. Given the typical number of participants of toddlers’ studies, the total number of samples within the database is expected to be small. Typical of this type of data, it is expected that different trials in toddlers’ VOE studies have similar duration—which is the time elapsed between the onset of a stimuli and the moment in which the toddler loses focus on the stimuli. If the duration of the trials is similar, and it is longer for valid trials—which is when toddlers are looking at the stimuli—and given the small dimensionality of the dataset, we should expect a classifier based on a Linear SVM to be suitable for the task. The limitations with this approach are that the assumption of linearity does not take into account the possibility that subsequent trials may be valid but have different duration, such as in the case in which one trial is significantly longer than the previous or subsequent one, nor does it take into account the effect of repetitions. While we can expect a novelty effect on the first trial, the effect will be reduced on subsequent trials. For these reasons, a SVM based on a non-linear kernel may provide better performances, as compared to a SVM classifier that employs a linear kernel. Additionally, given the nature of the data, we expect high similarity between valid and invalid trials between different participants in toddlers’ VOE studies. For this reason, it is well-founded to assume that to obtain a classifier that can be easily extended to future studies, a nonparametric classifier which is less influenced by autocorrelation and multicollinearity should be preferred over a parametric classifier [37]. Therefore, we decided to test a kNN classifier, which is a nonparametric instance-based learning classifier that can be effectively deployed and integrated with other tools.

### Aim and Hypothesis

In this work, we investigated the possibility of using ML models on toddlers’ fixation time data to automatically separate *usable* from *unusable* trials. More specifically, we hypothesise that novel machine learning models can be trained on human-labelled fixation signal trials to predict the usability of these trials at a greater than chance level.

## 2. Materials and Methods

### 2.1. Analytic Plan

**Figure 1:**
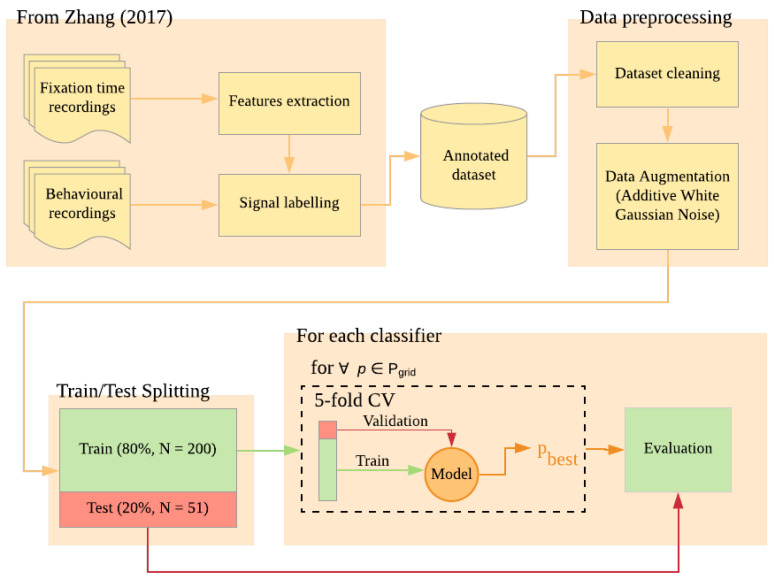
Summary of the steps employed for data augmentation, training, and hypertuning of the models.

In this work, fixation signals and their quality (*usable* vs. *unusable*) are drawn from a dataset collected previously for a study on young children’ expectations about the behaviours of leaders [38]. The dataset contains fixation data as well as an estimation of the signal usability made by the researcher based on both the signal and the behaviour of the children during the experimental sessions. Additional details about the original work, such as the sample size, are reported in Section 2.2. After dividing the available samples into two sets, Training and Test, the Training set is first processed using an Additive White Gaussian Noise (AVGN) data augmentation algorithm in order to increase the number of available samples and to balance the number of samples per class [39]. This is to avoid any possible bias due to the difference in the number of elements per class. Then, three different machine learning classifiers—a Liner SVC, a Non-Linear SVC, and a Nearest Neighbors Classifier—are tested to verify their performances in an automatic labelling task. A 80/20 Train/Test splitting is first performed, while a 5 fold cross validation is employed to verify the generalizability of the model. A visual representation of the overall process is displayed in Figure 1. The plan has been preregistered on the Open Science Framework (OSF), using the OSF preregistration template, which covers aspects of the sample size and demographics, data preprocessing, and ML analysis. The complete preregistration of this work can be found online at osf.io/ewy7z. Additionally, a statistical comparison of models’ performances is conducted by mean of a McNemar’s Test [40].

### 2.2. Data

The dataset employed in this work is drawn from a previous investigation conducted by Zhang [38]. In this work, three studies on toddlers’ expectations about leaders’ behaviour were conducted. In general, it was predicted that toddlers expect leaders to be fair and helpful but held no such expectations for nonleaders [41,42]. In Study 1, toddlers were presented with a scene in which the leader distributes two items to nonleaders with one of three outcomes: the leader is fair—she distributes two items equally to both nonleaders; the leader is unfair—she gives two items to one nonleader and none to the other nonleader; or the leader is selfish—she takes both items for herself and gives none to the nonleaders. If toddlers expect leaders to be fair as opposed to being selfish or unfair, they should look for longer fixation time at the unfair and selfish events. The longer looking time is interpreted as the events violating toddlers’ expectations. In Study 2, the leader either helps or is unhelpful to a nonleader. If children have the expectation that leaders should be helpful, they will look longer at the unhelpful than the helpful condition. Study 3 presented the same scenario, except that the roles of leader and nonleader were reversed. In Study 3, a nonleader either helps or is unhelpful toward the leader. If children expect leaders to be beneficient but have no such expectations of nonleaders, they will not find either the helpful or nonhelpful behaviour of the nonleader towards the leader unexpected. The protocol of the studies was approved by the Institutional Review Board of the Nanyang Technological University (IRB-NTU) and informed consent was obtained from the parents before each experimental session. Further details about the methodology employed in the original work can be found in Zhang [38].

The initial dataset used in the current work consisted of two hundred and fifty-one samples (N = 251) collected from one hundred and twelve participants (N = 112, Mean age = 25.7±5.3 months). Before each recording, the eye-tracking device was calibrated. After recording, a manual inspection of the signals was conducted by the researcher involved in the original study to assess the quality and usability of the recordings. This assessment was not exclusively based on the raw signal but also on observations made on the behaviour of the toddlers (e.g., *“Distracted and bored at the third video, turn around and fidget”*) [43]. During this phase, the signals were either labelled as **Usable** or **Unusable**. The breakdown of the sample into the two classes investigated (*Usable/Unusable*) for each study is reported in Table 1. The final dataset therefore consists of three features—Repetition 1, Repetition 2, Repetition 3—that correspond to the duration, in seconds, of each repetition of the trial. These three features are the independent variables used for Training and Testing of the model. The dependent target variable is a binary variable that indicates the usability of each Trial, that can be either **Usable** or **Unusable**. A copy of the dataset is available online on the data repository of the Nanyang Technological University (DR-NTU Data) [43].

### 2.3. Data Augmentation

Given the unbalanced number of samples per class, as well as the overall low number of samples, a data augmentation technique, Additive White Gaussian Noise (AVGN), was employed to increase the number of *Unusable* samples. This was also done to reduce the impact of the unbalanced number of samples per class on the training of the kNN classifier. The technique consisted of generating new synthetic samples by adding white noise to a copy of the whole or of a subset of the original sample [39,44,45]. The technique has been shown to be suitable for ML analysis and classification of different type of signals, including neurophysiological signals [27,46], and it has been successfully employed to enhance the accuracy of ML classifiers [39,47].

Here an AWGN (±1σ) [27] was applied to the features extracted from signals labelled as *unusable* in the Training set to balance the ratio between *Usable* and *Unusable* trials. The final dataset therefore consisted of three hundred and eighty three samples (N = 383, N = 200 *Usable* signals, N = 136 *Unusable* signals). A complete breakdown of the number of *Usable* and *Unusable* samples by set and study is reported in Table 2.

### 2.4. Classification

For classification, three different models were employed: a linear Support Vector Machine, a non-linear Support Vector Machine, and a K-Neighbour classifier. The models were implemented in Python, using the Numpy [48], Pandas [49], and Scikit-learn [50] packages and trained on the High-Performance Computing (HPC) Gekko Cluster of the Nanyang Technological University (Python v. 3.7.4, Numpy v. 1.17.4, Scikit-learn v. 0.21.3, Pandas v. 0.25.1). A 80/20 Train/Test split was performed, while a 5-fold cross validation was employed for the training of each model. Script employed for analysis, as well as the full parameters grid used for the hyperparameters’ tuning, can be found in the data repository of the Nanyang Technological University (DR-NTU Data) [43]. In addition to the classifiers’ accuracy, precision, and recall scores, the F1 scores and the Matthew Correlation Coefficients (MCC) measures are reported [51]. Accuracy, which is the ratio between correctly classified samples and the total number of samples, was biased for unbalanced dataset, as the metric provides an overoptimistic estimation of the classifier ability [52,53]. Multiple alternative metrics, such as the F1, which is the harmonic mean of precision and recall, has been widely employed in ML studies. Despite the general consensus toward the employment of F1 in both binary and multiclass classification task, F1 is not class-independent, meaning that it produces different results according to which class is labelled as positive and which is labelled as negative. Moreover, because F1 does not take into account samples correctly classified as negative recall scores, both the F1 scores and the Matthew Correlation Coefficients (MCC) measures are reported [51]. As an alternative measure to F1, the MCC, a special case of the ϕ coefficient, was introduced [54]. Introduced as a ML metric in 2000 [55], MCC takes into account all of the four confusion matrix categories (true positives, false negatives, true negatives, and false positives), as well as the proportion between positive and negative elements in the dataset. Moreover, the metric is not influenced by the assignment of positive and negative classes, meaning that it will produce consistent results when classes are swapped [56]. The MCC returns a value between −1 and +1, where a coefficient of +1 indicates a perfect prediction, 0 a prediction no better than chance, and −1 indicates a disagreement between observation and prediction. Therefore, the higher the MCC score, the better the performance of the model.

## 3. Results

Results of models’ performances on the validation—with the best set of parameters obtained during the hyperparameters tuning procedure—and on the test set are reported in Table 3. The best set of parameters, selected through the hyperparameter tuning phase, are reported in Appendix A.

Results show that all the employed classifiers achieved an accuracy above chance level (Accuracy >0.5). In terms of Accuracy and Recall on the test set, the Nearest Neighbors classifier outperformed the models based on Linear and Non-Linear SVC. A comparison of the models based on the McNemar’s test (Python 3.8.5, statsmodels 0.12.0), revealed significant differences in the performances of the Linear-SVC models with both the Non-Linear SVC ($\chi^{2}=7.0,\;p=1.07\times 10^{-6}$) and the K-neighbors ($\chi^{2}=7.0,\;p=1.21\times 10^{-7}$) classifiers; however, no significant differences are present between the performances of the Non-Linear SVC and the K-neighbors classifiers ($\chi^{2}=0.0,\;p=0.125$).

Additionally, the K-Neighbors classifiers scored higher on both the F1 Score and the MCC measures (F1 = 0.875, MCC = 0.493), as compared to the Linear (F1 = 0.676, 0.262) and Non-Linear SVC (F1 = 0.795, MCC = 0.492) Classifiers, as shown in Table 3.

For what concerns the importance of employed features, both an analysis conducted using a Permutation Features importance model on the best kNN estimator and an inspection of SVC’s coefficients reveals how the third repetition (*"Repetition 3"*) is the most predictive feature, followed by the second repetition (*"Repetition 2"*), and finally by the first (*"Repetition 1"*). Details about the results are reported in Appendix B.

## 4. Discussion

In this work, we tested the possibility of using different machine learning models to discriminate between *usable* and *unusable* fixation time signals collected from young children. Results of three models—a Linear SVC, a Non-Linear SVC, and a K-Neighbors, reported in Table 3 confirm the possibility of adopting ML models for the automatic classification of fixation time signals quality. More specifically, our results suggest that ML classifiers could be successfully employed to support researchers in the coding of the usability of toddlers’ fixation time recordings, for example in VOE studies. Performances of the different models, reported in Table 3, suggest that the Non-Linear SVC and and kNN are performing better than the Linear SVC Classifier. In our dataset, the binary classification has been conducted in such a way that the *usable* samples were labelled as positives, while *unusable* samples as negative. Therefore, Precision and Recall scores reflect respectively the ratio between True Positive, which are truly *usable* samples, against True and False positive, and True positive and False negative respectively [57].

Additionally, both the F1 scores and the MCC measures, which should be more accurate for binary classification [51], support the finding that the kNN classifier provides the best performances for the task despite not performing significantly differently from the Non-Linear SVC classifier. Overall, these results confirm our hypothesis that ML models can be employed to help researchers and clinicians automatically discriminate between usable and unusable fixation time recording. However, our results are not able to confirm, from a statistical point of view, that the kNN classifier is performing significantly better than the Non-Linear SVM Classifier. With regards to the adoption of one classifier versus another, some considerations should be thoughtfully evaluated, such as the computational power of employed machines, possibility of retraining the model continuously with the addition of new samples, and the possibility of implementing the model within eye-tracking software of hardware devices.

With regards to Precision and Recall, all of our classifiers performed better in Precision than Recall. Taken together, these findings suggest that there are a low number of False Positives (unusable samples identified as usable) identified by the models, but the classifiers were missing usable samples by classifying them as unusable. This bias led our model to be highly selective. By reducing the number of False Positives, the classifiers can be successfully employed with the assumption that, if a trial as been classified automatically as usable, it most probably is. This suggests that classifiers can produce datasets that contain almost exclusively usable samples and that can therefore be automatically used by non expert users or raters to rapidly generate a subset of usable samples for hypothesis testing, piloting, testing of new analysis algorithm, or for other purposes that require the selection of usable samples. On the other hand, the classifier was discarding some samples or trials that were labelled by the human researcher as usable. Given the nature of toddler studies, which are difficult to conduct and that are usually conducted on a small number of participants, maximising the number of usable samples becomes critical. We can conclude that models can be successfully employed to identify a subset of usable samples from the total sample, with very low risk of selecting unusable samples. At the same time, a manual screening on samples that have been deemed as unusable by the classifier could be necessary to increase the number of usable samples. Assuming a scenario like the one presented with our three studies, with a ratio of unusable samples between the 15% and 40%, will result in the necessity of manually inspecting only between the 30% and 45% of the full recordings, assuming the recall rate of the kNN Classifier (Recall = 0.795). Overall, this results in a large reduction of the time necessary to manually label a collection of recordings.

Our findings can be useful for the development of smart tools to support researchers and especially coders working with behavioural and signal quality in young children’s fixation time studies. Future works may replicate the analysis with other types of pupillary measures, such as the pupil size, and may investigate the possibility of integrating a tool based on ML models directly into eye-tracking devices’ recording software. Future studies may investigate the possibility of adopting Cloud-Based Machine Learning Models to deploy the model as a software that can be integrated into different data analysis pipelines [58,59] or to devices connected to the Internet [27].

### Limitations

Despite these promising results, this study has some limitations. The dataset here used for training and testing of the models was based on three different studies recorded with the same instruments, and it contained a limited number of samples. Future studies should replicate this analysis by training and testing the models on more samples, collected from multiple projects. This would also allow for the testing of different classifiers that perform better on datasets containing higher number of samples, such as Decision Tree Classifiers. Moreover, our models are based on features already extracted and have no knowledge about the setup and the participants. Future studies may investigate the possibility of using Artificial Intelligence model that mingles estimated features with video recordings of the sessions to improve the accuracy of the models in automatically separating *Usable* from *Unusable* trials.

## 5. Conclusions

In this work, we investigated the possibility of using machine learning models to automatically separate *Usable* from *Unusable* toddlers’ fixation time samples.

Although the data were originally collected for a different purpose, and despite the relatively small number of samples available, our classifiers obtained promising results. We envision the possible adoption of ML models to support researchers in the manual preprocessing and labelling of collected fixtation time data.

Future studies should verify whether other models can outperform the models here trained by employing larger samples obtained by different studies, as well as testing different sets of features and measures, such as pupil size measures.

## Figures and Tables

**Table 1:** Participants’ demographic information and number of usable and unusable samples per Study included in this work.

Study	Participants	Age (Months)	*Usable* Samples	*Unusable* Samples	Total Number of Samples
Study 1	48	24.6 ± 4.5	71	26	97
Study 2	32	26.9 ± 4.9	60	17	77
Study 3	32	26.7 ± 6,2	69	8	77

**Table 2:** Usable and Unusable Samples in Training and Test Score by Experiment.

Study	Training Set			Test Set	
	Usable	Unusable	Unusable after AGWN	Usable	Unusable
Study 1	57	21	84	14	5
Study 2	47	15	13	13	2
Study 3	52	8	32	17	0

**Table 3:** Model’s scores (Accuracy, Precision, Recall, F1, and MCC) by type of classifier and test set.

Classifier	Train_MCC	Train_Acc.	Accuracy	Precision	Recall	F1	MCC
Linear SVC	0.207	0.602	0.576	0.958	0.523	0.676	0.262
Non-Linear SVC	0.491	0.810	0.706	1.00	0.659	0.795	0.492
K-neighbors	0.533	0.810	0.803	0.972	0.795	0.875	0.493

**Figure A1:**
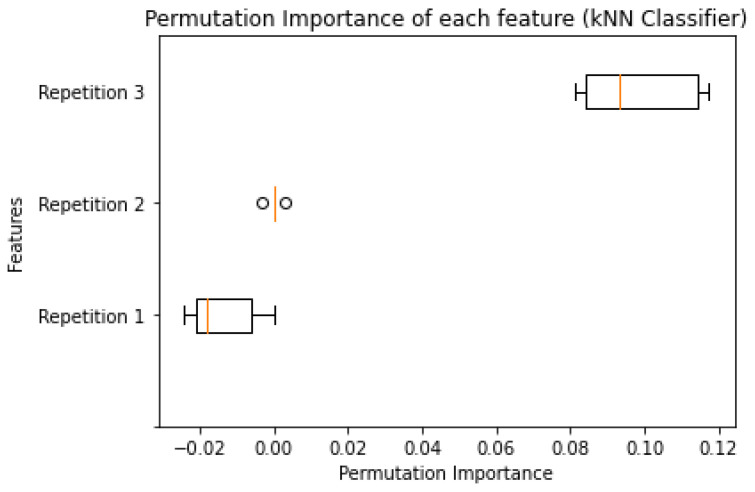
kNN features predictive weight (*Importance Mean*) obtained through permutation feature importance.

**Figure A2:**
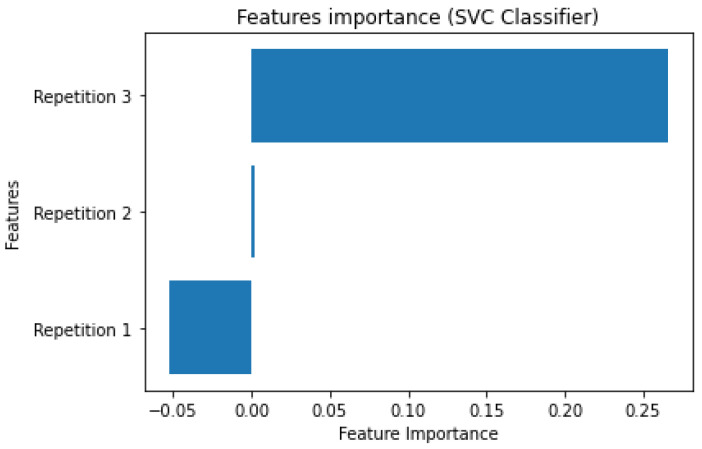
SVM features predictive weight.

## Abbreviations

The following abbreviations are used in this manuscript:

VOE	Violation-of-Expectation
ML	Machine Learning
AWGN	Additive White Gaussian Noise
SVC	Support Vector Machine
kNN	Nearest Neighbors
HPC	High-Performance Computer
MCC	Matthews Correlation Coefficient

## Appendix A. Models’ Parameters

Models have been trained and tested in Scikit-learn v. 0.21.3. After hypertuning, the models with the best parameters used for testing were:

**Table A1:** Best hyper-parameters for the trained models.

Classifier	Best Parameters
Linear SVC	‘C’: 30, ‘random_state’: 1, ‘tol’: 0.1
Non-Linear SVC	‘C’: 40, ‘decision_function_shape’: ‘ovo’, ‘gamma’: ’scale’, ‘kernel’: ‘rbf’, ‘random_state’: 0, ‘tol’: 0.1
K-Neighbors	‘leaf_size’: 1, ‘n_neighbors’: 8

## Appendix B. Features’ Relevance

### Appendix B.1. kNN

A permutation feature importance conducted on the best estimator of the kNN classifier is here reported.

**Table A2:** kNN features predictive weight (*Importance Mean*) obtained through permutation feature importance.

Repetition 1	Repetition 2	Repetition 3
−0.0151	0	0.1265

### Appendix B.2. SVM

Features importance based on our SVM best estimator’s coefficient are here reported and illustrated.

**Table A3:** SVM features’ predictive weights.

Repetition 1	Repetition 2	Repetition 3
−0.05269684	0.00185775	0.26624044

## References

[1] Hepach R., Westermann G. (2016). Pupillometry in infancy research. J. Cogn. Dev..

[2] Cashon C.H., Cohen L.B. (2000). Eight-month-old infants’ perception of possible and impossible events. Infancy.

[3] Cohen L.B., DeLoache J.S., Pearl R.A. (1977). An examination of interference effects in infants’ memory for faces. Child Dev..

[4] Fantz R.L. (1964). Visual experience in infants: Decreased attention to familiar patterns relative to novel ones. Science.

[5] Woodward A.L. (1998). Infants selectively encode the goal object of an actor’s reach. Cognition.

[6] Setoh P., Wu D., Baillargeon R., Gelman R. (2013). Young infants have biological expectations about animals. Proc. Natl. Acad. Sci. USA.

[7] Spelke E.S., Breinlinger K., Macomber J., Jacobson K. (1992). Origins of knowledge. Psychol. Rev..

[8] Sodian B., Schoeppner B., Metz U. (2004). Do infants apply the principle of rational action to human agents?. Infant Behav. Dev..

[9] Baillargeon R., Scott R.M., He Z., Sloane S., Setoh P., Jin K.S., Wu D., Bian L. (2015). Psychological and Sociomoral Reasoning in Infancy.

[10] Kobayashi T., Hiraki K., Mugitani R., Hasegawa T. (2004). Baby arithmetic: One object plus one tone. Cognition.

[11] Onishi K.H., Baillargeon R. (2005). Do 15-month-old infants understand false beliefs?. Science.

[12] Luo Y., Kaufman L., Baillargeon R. (2009). Young infants’ reasoning about physical events involving inert and self-propelled objects. Cogn. Psychol..

[13] Méary D., Kitromilides E., Mazens K., Graff C., Gentaz E. (2007). Four-day-old human neonates look longer at non-biological motions of a single point-of-light. PLoS ONE.

[14] Bangee M., Harris R.A., Bridges N., Rotenberg K.J., Qualter P. (2014). Loneliness and attention to social threat in young adults: Findings from an eye tracker study. Personal. Individ. Differ..

[15] Aslin R.N., McMurray B. (2004). Automated corneal-reflection eye tracking in infancy: Methodological developments and applications to cognition. Infancy.

[16] Libby W.L., Lacey B.C., Lacey J.I. (1973). Pupillary and cardiac activity during visual attention. Psychophysiology.

[17] Mock J., Huber S., Klein E., Moeller K. (2016). Insights into numerical cognition: Considering eye-fixations in number processing and arithmetic. Psychol. Res..

[18] Ruba A.L., Johnson K.M., Harris L.T., Wilbourn M.P. (2017). Developmental changes in infants’ categorization of anger and disgust facial expressions. Dev. Psychol..

[19] Hamlin J.K., Wynn K. (2011). Young infants prefer prosocial to antisocial others. Cogn. Dev..

[20] Jin K.S., Houston J.L., Baillargeon R., Groh A.M., Roisman G.I. (2018). Young infants expect an unfamiliar adult to comfort a crying baby: Evidence from a standard violation-of-expectation task and a novel infant-triggered-video task. Cogn. Psychol..

[21] Premack D., Premack A.J. (1997). Infants attribute value± to the goal-directed actions of self-propelled objects. J. Cogn. Neurosci..

[22] Tafreshi D., Thompson J.J., Racine T.P. (2014). An analysis of the conceptual foundations of the infant preferential looking paradigm. Hum. Dev..

[23] Hamlin J.K., Wynn K., Bloom P. (2007). Social evaluation by preverbal infants. Nature.

[24] Peterson D. (2016). The baby factory: Difficult research objects, disciplinary standards, and the production of statistical significance. Socius.

[25] Rubio-Fernández P. (2019). Publication standards in infancy research: Three ways to make Violation-of- Expectation studies more reliable. Infant Behav. Dev..

[26] Pickering A. (2010). The Mangle of Practice: Time, Agency, and Science.

[27] Gabrieli G., Bornstein M.H., Manian N., Esposito G. (2020). Assessing Mothers’ Postpartum Depression From Their Infants’ Cry Vocalizations. Behav. Sci..

[28] Li Q., Clifford G.D. (2012). Dynamic time warping and machine learning for signal quality assessment of pulsatile signals. Physiol. Meas..

[29] Li Q., Rajagopalan C., Clifford G.D. (2014). A machine learning approach to multi-level ECG signal quality classification. Comput. Methods Programs Biomed..

[30] Pauly L., Sankar D. Detection of Drowsiness Based on HOG Features and SVM Classifiers. Proceedings of the 2015 IEEE International Conference on Research in Computational Intelligence and Communication Networks (ICRCICN).

[31] Kang J., Han X., Song J., Niu Z., Li X. (2020). The identification of children with autism spectrum disorder by SVM approach on EEG and eye-tracking data. Comput. Biol. Med..

[32] Dalrymple K.A., Jiang M., Zhao Q., Elison J.T. (2019). Machine learning accurately classifies age of toddlers based on eye tracking. Sci. Rep..

[33] Wang S., Jiang M., Duchesne X.M., Laugeson E.A., Kennedy D.P., Adolphs R., Zhao Q. (2015). Atypical visual saliency in autism spectrum disorder quantified through model-based eye tracking. Neuron.

[34] Zhang Y., Bulling A., Gellersen H. (2011). Discrimination of gaze directions using low-level eye image features. Proceedings of the 1st International Workshop on Pervasive Eye Tracking & Mobile Eye-Based Interaction, Septmber 2011.

[35] Kacur J., Polec J., Csóka F. Eye Tracking and KNN Based Detection of Schizophrenia. Proceedings of the 2019 International Symposium ELMAR.

[36] Vernon P.E. (1933). The rorschach ink-blot test 1. I. Br. J. Med. Psychol..

[37] Murphy K.P. (2012). Machine Learning: A Probabilistic Perspective.

[38] Zhang L. (2017). Infants’ Moral Expectations about Authority Figures. Ph.D. Thesis.

[39] Rochac J.F.R., Zhang N., Xiong J., Zhong J., Oladunni T. Data Augmentation for Mixed Spectral Signatures Coupled with Convolutional Neural Networks. Proceedings of the 2019 9th International Conference on Information Science and Technology (ICIST).

[40] Dietterich T.G. (1998). Approximate statistical tests for comparing supervised classification learning algorithms. Neural Comput..

[41] Stavans M., Baillargeon R. (2019). Infants expect leaders to right wrongs. Proc. Natl. Acad. Sci. USA.

[42] Stavans M., Diesendruck G. (2020). Children Hold Leaders Primarily Responsible, Not Entitled. Child Dev..

[43] Gabrieli G., Zhang L., Setoh P. (2020). Related Data for: “A Machine Learning Approach for the Automatic Estimation of Fixation-Time Data Signals’ Quality”.

[44] Antoniou A., Storkey A., Edwards H. (2017). Data augmentation generative adversarial networks. arXiv.

[45] Sarma B.D., Dey A., Lalhminghlui W., Gogoi P., Sarmah P., Prasanna S. Robust Mizo digit recognition using data augmentation and tonal information. Proceedings of the 9th International Conference on Speech Prosody.

[46] Um T.T., Pfister F.M., Pichler D., Endo S., Lang M., Hirche S., Fietzek U., Kulić D. Data augmentation of wearable sensor data for parkinson’s disease monitoring using convolutional neural networks. Proceedings of the 19th ACM International Conference on Multimodal Interaction.

[47] Bjerrum E.J., Glahder M., Skov T. (2017). Data augmentation of spectral data for convolutional neural network (CNN) based deep chemometrics. arXiv.

[48] Walt S.V.D., Colbert S.C., Varoquaux G. (2011). The NumPy array: A structure for efficient numerical computation. Comput. Sci. Eng..

[49] McKinney W. (2011). pandas: A foundational Python library for data analysis and statistics. Python High Perform. Sci. Comput..

[50] Pedregosa F., Varoquaux G., Gramfort A., Michel V., Thirion B., Grisel O., Blondel M., Prettenhofer P., Weiss R., Dubourg V. (2011). Scikit-learn: Machine learning in Python. J. Mach. Learn. Res..

[51] Chicco D., Jurman G. (2020). The advantages of the Matthews correlation coefficient (MCC) over F1 score and accuracy in binary classification evaluation. BMC Genom..

[52] Sokolova M., Japkowicz N., Szpakowicz S. (2006). Beyond accuracy, F-score and ROC: A family of discriminant measures for performance evaluation. Proceedings of the Australasian Joint Conference on Artificial Intelligence.

[53] Bekkar M., Djemaa H.K., Alitouche T.A. (2013). Evaluation measures for models assessment over imbalanced data sets. J. Inf. Eng. Appl..

[54] Guilford J.P. (1954). Psychometric Methods.

[55] Baldi P., Brunak S., Chauvin Y., Andersen C.A., Nielsen H. (2000). Assessing the accuracy of prediction algorithms for classification: An overview. Bioinformatics.

[56] Gorodkin J. (2004). Comparing two K-category assignments by a K-category correlation coefficient. Comput. Biol. Chem..

[57] Powers D.M. (2011). Evaluation: From Precision, Recall and F-Measure to ROC, Informedness, Markedness and Correlation. arXiv.

[58] Gabrieli G., Azhari A., Esposito G. (2020). PySiology: A python package for physiological feature extraction. Neural Approaches to Dynamics of Signal Exchanges.

[59] Bizzego A., Battisti A., Gabrieli G., Esposito G., Furlanello C. (2019). pyphysio: A physiological signal processing library for data science approaches in physiology. SoftwareX.

